# Cerebrovascular Pressure Reactivity in Children with TBI

**DOI:** 10.15844/pedneurbriefs-29-10-4

**Published:** 2015-10

**Authors:** Laurence Ducharme-Crevier

**Affiliations:** 1 Ruth D. & Ken M. Davee Pediatric Neurocritical Care Program, Ann & Robert H. Lurie Children's Hospital of Chicago, Chicago, IL; 2 Departments of Pediatrics and Neurology, Northwestern University Feinberg School of Medicine, Chicago, IL

**Keywords:** Pediatric, Traumatic Brain Injury, Cerebral Autoregulation

## Abstract

Investigators from University of Melbourne, Australia, studied Pressure-Reactivity Index (PRx) and optimal Cerebral Perfusion Pressure (CPP) in 36 children aged between 6 months and 16 years treated for traumatic brain injury (TBI) at the Royal Children's Hospital, Melbourne, from 2007 to 2013.

Investigators from University of Melbourne, Australia, studied Pressure-Reactivity Index (PRx) and optimal Cerebral Perfusion Pressure (CPP) in 36 children aged between 6 months and 16 years treated for traumatic brain injury (TBI) at the Royal Children's Hospital, Melbourne, from 2007 to 2013. All patients received care according to the local TBI protocol. Patients were monitored for a median of 3.8 days. The authors described PRx in this cohort of children with TBI and examined its association with other patient variables. They also compared patients in the favorable and unfavorable outcome groups. PRx quantifies the correlation between intracranial pressure (ICP) and arterial blood pressure to assess the integrity of cerebral blood flow autoregulation. CPP refers to the difference between mean arterial pressure and ICP. Optimal CPP is the ideal pressure gradient driving cerebral perfusion in light of the evolution of PRx over time for each patient.

PRx was significantly higher in children with unfavorable outcome (p<0.01). Therefore, loss of cerebral autoregulation was associated with worse outcome. The ideal PRx threshold to discriminate between favorable and unfavorable outcome is still unknown, but a threshold PRx value of both 0 and 0.25 seem to delineate between a favorable and an unfavorable outcome (both thresholds, p<0.01). Nonetheless, PRx is a useful prognostic marker.

In line with previous adult studies, the relationship between the PRx and CPP respected a U-shape curve, allowing detection of an optimal CPP for each patient. The optimal CPP varied significantly with age. In this cohort of patients the optimal CPP ranged from 53 to 78 mm Hg. Furthermore, the patients with unfavorable outcome spent a greater percentage of their monitoring time with a CPP at least 5 mm Hg inferior to their optimal CPP. [1]

COMMENTARY. The prognostic role of PRx has been established in adult studies, but the concept is relatively new in the pediatric population [2]. An impaired cerebral autoregulation increases the risk of poor outcome. This study confirms the utility of PRx in children with TBI to discriminate outcome.

Secondary brain injuries are the result of events following the initial TBI. Avoidance of further secondary insult to the injured brain is the basis of TBI management. The Guidelines for the acute medical management of severe pediatric TBI suggest a CPP threshold of 40 to 50 mm Hg to avoid secondary ischemic injury, with the understanding that age-specific threshold probably exists, with infants at the lower end and adolescents at the higher end of this range [3]. This current study underlines the increase of optimal CPP with age. Age-specific and individualized CPP targeting may be the future of TBI management as patients of similar age demonstrate different optimal CPP. This may partly explain why targeting a specific CPP number for all patients have not been proven successful [4].

In summary this article emphasizes the potential value of PRx monitoring as both a prognostic marker and a determinant of individualized optimal CPP. The potential therapeutic role of individualized care for patient and optimal CPP targeting based on PRx evaluation need further clinical evaluation to assess the clinical impact on patient outcome [5].
